# Clinic Atlanta Medical College

**Published:** 1874-04

**Authors:** E. A. Cobleigh

**Affiliations:** Reporter


### EYE AND EAR.

*A. W. Calhoun, M.D., Professor of Diseases of the Eye and Ear.*

E. A. COBLEIGH, M.D. Reporter.

Case 16.—January 3d. Benj. G., colored, school-teacher from Newnan, Ga., æt. twenty-three years. This patient had an opacity of the cornea of both eyes, the opacity being located directly over pupil in the left. In right eye there was also found to exist an anterior synechia. Cause of opacities, ulcerative corneitis, which occurred several years ago, with perforation in right eye. Treatment—an iridectomy was made on left eye to give him use of that member by the artificial pupil. 7th. He sees already some better, but the inflammation resulting from operation has not yet subsided enough to give clear vision. As he insisted on now returning home, he was discharged with excellent prospects for a speedy and satisfactory recovery.

J. W. P., student of the class, twenty-one years old. Came for treatment January 10th, suffering from chronic blepharadinitis. Ordered to use the following ointment twice daily on the edges of eye-lids after thoroughly cleansing them:

℞.—Hydrarg. Oxidi Rub., . . . . grs. ij.
Unguent. Aquæ Rosæ . . . . ℥ j.—M.

February 1st, recovered and was discharged.

Case 18.—January 14th.. Caroline C., æt. thirty-eight years, white. Came before the class with granular lids.

℞.—Argenti Nitratis . . . . . . grs. x.
Aquæ Puræ . . . . . . . f. ℥ j.

Misce. Apply to lids once or twice a day. February 4th. Improved, especially the right eye. Continue treatment. February 11th. Still improving, and ordered to persevere in the use of the foregoing solution. Did not present herself at the clinic any more, and therefore no further note of the progress of the case could be taken.

Case 19.—January 17th. William B., a white laborer, sixty-nine years old. This man applied for treatment, suffering the same as the last mentioned patient, and was put upon the same remedy; case progressing to speedy recovery and patient being discharged well.

Case 20.—Chas. R., æt. thirteen years, white. January 20th. Examination revealed morgagnian cataract (which his history proved was congenital) in both eyes. The treatment was by discission. February 4th. Lens in left eye is nearly absorbed; in right has completely disappeared except posterior capsule. Prof. Calhoun at once broke up the capsule in both eyes. February 7th. Can see, but cannot distinguish objects, except by touch, as he never had any vision heretofore. February 11th. Posterior capsule alone remains in right eye; but a small portion of lens is yet unabsorbed in the left. Pupils have been kept constantly dilated with atropine. February 16th. Lens of left eye nearly disappeared, remaining portion being again divided by the Professor. February 21st. To-day there was ordered for him a solution of strychnia ($\frac{1}{40}$ gr. to 6 gtts. water), the same to be injected every day, and gradually increased after several weeks.

Case 21.—January 21st. Terrell R., white, eight years of age, of delicate health. This child also had congenital cataract of both eyes, and was treated as the preceding case. February 4th. Absorption of lenses had been slow and the operation of discission was repeated. February 7th. Both eyes somewhat inflamed; right one not doing so well as the left, and there now is an opening into the posterior chamber. February 11th. Right lens has been absorbed, but capsule remains and is very tough. Absorption proceeds very tardily in the other eye. Ordered to be put upon injections of strychnia in minute doses ($\frac{1}{30}$ gr. to begin on) and gradually increased, the injections to be made in the temples. February 16th. Patient was anæsthetized and eyes again operated upon, but in the right the needle, on account of the toughness of capsule, was introduced through the sclera. Case went on, after this, to recovery, with little more trouble.

Case 22.—January 28th. Eveline B., washerwoman, æt. forty-eight, from Stewart county. Left eye began itching violently six years ago, and became much inflamed. One year back vision was lost altogether in the left eye, and the other one became affected. Sharp, keen pains occasionally dart through the left eye, and it still remains inflamed. Posterior synechiæ in both eyes, probably the result of iritis. Her general health is good, but menstruation occurs every fourteen days. Diagnosis: Secondary cataract. Dr. C. undertook the operation, having first, however, informed the patient that this step would probably not restore her sight, but only destroy the tendency to iritis. After extraction of the cataract of the left eye, she was able to distinguish near objects around her. February 4th. Right eye was operated upon as had been its fellow. Could then count the fingers with that eye also, and tell one from another. February 7th. Case rapidly improving, and cure without unpleasant symptoms almost certain.

Case 23.—S. W. L., white, a member of the medical class, twenty-two years of age. Presented himself for examination on January 31st. Traumatic cataract exists in one of his eyes, but does not want it treated. Is suffering from myringitis, the inflammation having extended to the meatus auditorious externus:

| | |
|---|---|
| ℞.—Belladonæ (Ext.), . . . . . | grs. xvj. |
| Hydrarg. Precip. Albæ, . . . | grs. xij. |
| Morphiæ Sul. . . . . . | grs. ij. |
| Unguent. Aquæ Rosæ, . . . | ℨ. ij.—Misce. |

Sig. Rub around the affected ear three times a day. Also use milk-warm water and laudanum in the meatus three or four times daily. Recovered in a short time.

Case 25.—February 4th. Louis G., æt. twenty-two years, colored. Has had some disease of the eye resulting in deep ulcer of cornea, leaving the upper third or half of that membrane opaque. Treatment: an iridectomy for the formation of new pupil one side of the opacity. February 7th. The inflammation has nearly disappeared. February 11th. Eye well, sees nicely and has good artificial pupil.

Case 26.—February 21st. Lavenia L., negress, æt. fifteen years. This girl took cold a few days ago, and now not only has lancinating pains in left ear but can hear nothing with that ear at all. Treatment: Pour a teaspoonful of sol. bicarb. soda (ʒ j. to water f. ℥ ss.) into the ear thrice daily and let it remain five minutes, then plug the meatus with cotton. Return Monday for more thorough examination. February 25th. Examined, and acute otitis media found to exist, as was suspected when here before. Continued treatment. A purgative was also ordered for to-night. February 25th. Great improvement, but now complains of roaring in ear. Put upon opium and quinine. Recovered in short time.

Case 29.—Peter K., German, æt. about forty years, from Alabama. This man came before the class on February 16th, and gave as the history of his case that vision became very much impaired four years ago, but by long treatment, was partially restored. Since two years ago vision has again been failing, and six months back he became totally blind. A little before the first attack patient says he contracted syphilis, and was suffering from its effects when the sight became impaired, but all unpleasant symptoms disappeared simultaneously with recovery of vision. Opthalmoscopic examination at present reveals the vitreous humor of both eyes very opaque, with large bodies floating about in it in all directions. In the left eye the vitreous was clear enough to admit of close scanning of the optic-disk which presented that peculiar whitish appearance indicative of atrophy of optic nerve, and all the blood-vessels running out over disk were much contracted. In the right eye the vitreous was too opaque to allow satisfactory inspection of the disk, but it was presumable that it was in condition similar to the other. The man complained of excruciating pain in frontal region, precluding any idea of rest at night. Otherwise patient is in usually good health. On testing vision he was only able to describe movement of the hand before the eye, as the passing of some large body, but could not recognize the hand itself. Treatment—Commenced the hypodermic use of strychnia in the temples, once a day, using a solution of $\frac{1}{40}$ grain to 6 drops water, and gradually increasing amount of the strychnia. March 15th, he was receiving $\frac{1}{10}$ gr. strychnia at each injection. Mercurial inunctions were begun at the same time as the strychnia, and were used once daily ( ʒj. each time) alternating with different parts of the body. After the sixth inunction salivation was produced and the mercurial stopped, no other remedy being used at all up to present time except the strychnia hypodermically. The frontal pains ceased after the fourth day's treatment and have not returned ; the appetite and general strength also increased rapidly; vision improved after the first week, and to-day he is able to slowly read Schnellen's test types No. 30, with accuracy, at four feet distance, and most of the letters of No. 20 at three feet. He now recognizes objects on the streets, reading large letters on sign-boards and placards, and can, across the street, distinguish a negro from a white person. Examination with the opthalmoscope shows the vitreous of both eyes rapidly clearing up, and admits, in the left one, of a thorough inspection of the disk which still maintains atrophic appearance with the contracted vessels. The treatment used will be persevered in with steady increase of amount of strychnia each day for some time to come.

Case 31.—March 11th. John C., freedman, forty years of age, from Lumpkin county, Ga. Patient has had frequent attacks of iritis in both eyes, the inflammation in the right one extending to ciliary bodies. As a result of the inflammatory process there is in both eyes complete occlusion of the pupil by exudative membrane filling up the space. Complete circular adhesions (synechiæ) have taken place so that very strong solutions of atropine do not dilate the pupils in the least. In consequence of a deep-seated inflammation in the right eye total opacity of the lens has been brought about. In the left one it could not be positively ascertained whether the lens was opaque or not on account of the peculiar position of the pupilary membrane. Operation: Large iridectomy was made to upper and outer part of iris in left eye, after which lens was found quite clear, exudative membrane simply covering the center; patient could count the fingers immediately. In the right eye Dr. C. extracted the cataract (after making a very large iridectomy) by Graafe's linear method, taking out at the same time the adventitious membrane as nearly entire as possible. The patient could then also distinguish and count fingers with this eye. [Before the operation, in testing vision, he had simply good perception of light, but was unable to recognize objects in the least.] 15th. In the interval eyes have been dressed with ordinary bandages twice daily, after thoroughly cleansing from all secretions. The man is doing well in all respects, and the operation bids fair to prove a perfect success.

---

A Norristown doctor recently asked an old lady patient if she experienced any relief during the night. She said she did. First, the relief was in one shoulder and then the other, and then 'peared to settle in her back, but she put a mustard plaster between her shoulders, and the relief left her, and now she feels better.

